# Progress in Fevers

**Published:** 1903-07-18

**Authors:** 


### Progress in Fevers.

(*Concluded from page* 263.)

**Typhoid Fever** (*continued*).—Hale White [14] in a clinical lecture delivered at Guy's Hospital touches on a few of the difficulties connected with the differential diagnosis of typhoid fever as illustrated by some patients. In the case of a girl aged 14 diagnosis lay between typhoid fever and appendicitis. The points on which stress was laid in diagnosing the latter disease were a sudden onset, considerable abdominal pain, considerable—although general not localised—abdominal tenderness, and vomiting. In typhoid onset is gradual, abdominal pain and tenderness are not complained of, and vomiting is not marked. Headache, in this case transient, is in typhoid a more persistent and severe frontal ache. The blood count is very important. Contrary to the general rule in the specific fevers, in typhoid there is leucopenia not leucocytosis; the white corpuscles may fall from the normal count of 10,000 per c.mm. to as few as 1,000 per c.mm. If leucocytosis occurs it indicates the onset of some complication of which the commonest is phlebitis. In appendicitis, as in other diseases, leucocytosis occurs directly pus begins to be formed, and even before that event takes place in all except the most trivial cases. The resemblance to meningitis may also be close, and a case is cited where a boy aged eight showed marked head retraction, a curled-up attitude, and great intolerance of interference, together with raised pulse and temperature. Thorough examination revealed an enlarged spleen and *one* typhoid spot. Great stress is laid by Hale White on the value of typhoid spots in diagnosis. The spots are pale red, roundish, vary in size from a pin-head to a large pea, are slightly elevated, lose their colour on pressure, and appear in crops. They usually appear from about the seventh to the tenth day, chiefly on the trunk, and are practically never hæmorrhagic. Other points to note in distinguishing meningitis from typhoid fever are the greater severity of headache in meningitis and its continuance during delirium, unequal or minutely contracted pupils, unequal reddening of the discs, and retraction of the abdomen. In a series of 95 cases of enteric under the care of J. H. Bellamy [15] a rash was observed in only 39 cases, and in two of these it was morbilliform in character, tended to form crescents, and appeared in one single crop. Among the complications of enteric fever recently recorded is a case of symmetrical gangrene of both lower limbs, ascribed to arterial thrombosis involving the aorta at its bifurcation, under the care of C. E. Nammack.[16] Lewis and Le Conte have described two cases in which suppuration in an ovarian cyst occurred during defervescence. Typhoid bacilli in pure culture were found in the pus. Meningeal complications may greatly hamper the diagnosis of enteric.[17] Cases with cerebro-spinal symptoms in which Kernig's sign is present stand Brand's cold method of treatment badly. Hödlmoser [18] has observed severe convulsions in a man, aged 40, who recovered. Cases of hæmorrhagic typhoid fever are reported by Kingston Fowler and Foulerton,[19] and a case of "nephro-typhoid" fever by Ledingham and McCrorie,[20] in which renal symptoms appeared early and predominated. In the *treatment* of typhoid fever Hale White [21] discusses, first, the dietetic treatment, and points out that provided that the food is easily digested and absorbed, and such that solid or irritating particles cannot reach the ileum, the necessary conditions are fulfilled. Milk, which most nearly fulfils these conditions, he still regards as the staple food, especially for hospital patients, where "visitors" and their gifts have to be contended with. Being a fluid diet milk is also desirable for patients suffering from high and prolonged fever, dry mouth, and inability to masticate properly. Milk is deficient in carbohydrate, the proteid-sparing properties of which are valuable in fevers. The deficiency may be made up by adding lactose or maltine to the milk. Two ounces of the latter may be given to an adult every 24 hours. If diarrhœa occurs examine the stools for curds, and peptonise the milk to prevent their occurrence. Alcohol is only valuable if, as sometimes does happen in fevers, it slows the pulse-rate. It is not suitable for sudden collapse. Bathing or sponging should not be employed as a routine, but should be used only where the patients are obviously suffering from the high fever. Perforation and profuse hæmorrhage are the only contra-indications. Bronchitis appears to be actually benefited. The bath should never be lowered below 70° F., nor continued after the rectal temperature has fallen to 100°. For warding off the cardiac failure so common towards the end of the third week Hale White gives 12 to 15 minims of tincture of digitalis every four hours, with caffeine and strychnine. B. Baskett [22] has found antityphoid serum very beneficial as a curative agent in four cases in which he has tried it. M. M. Bernstein[23] strongly recommends the use of the common bilberry or whortleberry (Vaccinium myrtillus) as an intestinal antiseptic, astringent, and antifermentative. The jam or infusion may be given freely, as it is non-poisonous. In laboratory experiments carried out with a watery decoction of the juice B. typhosus and B. coli communis were found to be killed after 24 to 48 hours in the acid juice, and before 24 hours in the neutralised juice. H. P. Loomis[24] has analysed the typhoid fever returns in the four New York hospitals for the last two years. The mortality works out at 10 per cent. in each. Summarising the treatment in these hospitals, he finds milk, peptonised or with lime-water, is the only diet until the temperature is either normal or below 100°, when soft-boiled eggs, chicken, beef sandwiches, and chops are added in rapid succession. Whisky in half-ounce doses is used as a stimulant. The Brand bath is given when the temperature reaches 103°. Trional is used for sleeplessness. For tympanites the ice coil or turpentine stupes are used, and turpentine in 5 ♏ doses is given internally. Majendie hypodermically is given for hæmorrhage.

[14] Clin. Jour., Dec. 17. [15] Quart. Med. Jour., May. [16] Med. Rec., Dec. 27. [17] Treatment, Dec. [18] Brit. Med. Jour. Epitome, Jan. 10. [19] Ibid., Jan. 3. [20] Lancet, Feb. 28, p. 587, and Brit. Med. Jour., Feb. 28, p. 492. [21] Brit. Med. Jour., Feb. 14, p. 368. [22] Clin. Jour., Dec. 24. [23] Brit. Med. Jour. Feb. 21, p. 423. [24] Brit. Med. Jour., Feb. 7, p. 306. [25] Med. Rec. Jan. 10.

